# Phase II study of the immune-checkpoint inhibitor ipilimumab plus dacarbazine in Japanese patients with previously untreated, unresectable or metastatic melanoma

**DOI:** 10.1007/s00280-015-2870-0

**Published:** 2015-09-25

**Authors:** N. Yamazaki, H. Uhara, S. Fukushima, H. Uchi, N. Shibagaki, Y. Kiyohara, A. Tsutsumida, K. Namikawa, R. Okuyama, Y. Otsuka, T. Tokudome

**Affiliations:** Department of Dermatologic Oncology, National Cancer Center Hospital, Tokyo, Japan; Department of Dermatology, Shinshu University School of Medicine, Matsumoto, Japan; Department of Dermatology and Plastic Surgery, Faculty of Life Sciences, Kumamoto University, Kumamoto, Japan; Department of Dermatology, Graduate School of Medical Sciences, Kyushu University, Fukuoka, Japan; Department of Dermatology, Yamanashi University Hospital, Yamanashi, Japan; Dermatology Division, Shizuoka Cancer Center, Shizuoka, Japan; Research and Development, Bristol-Myers K.K., 6-5-1, Nishishinjuku, Shinjuku-ku, Tokyo, 163-1328 Japan

**Keywords:** Ipilimumab, Dacarbazine, Immune-checkpoint inhibitor, Melanoma, Phase 2 study, Japanese patients

## Abstract

**Purpose:**

Ipilimumab (IPI), a monoclonal antibody against immune-checkpoint receptor cytotoxic T lymphocyte antigen-4, is designed to enhance antitumor T cell function. IPI 10 mg/kg plus dacarbazine (DTIC) significantly improved overall survival in a phase 3 study involving predominantly Caucasian patients, with an adverse event (AE) profile similar to that of IPI monotherapy. We conducted a single-arm, phase 2 study to evaluate the safety and efficacy of IPI plus DTIC in Japanese patients.

**Methods:**

Previously untreated patients with unresectable stage III or IV melanoma received IPI 10 mg/kg plus DTIC 850 mg/m^2^ every 3 weeks for four doses (q3w × 4), followed by DTIC q3w × 4 and then IPI every 12 weeks until disease progression or intolerable toxicity.

**Results:**

All 15 treated patients reported drug-related AEs, the most common of which were increases in alanine aminotransferase (*n* = 12, 80 %) and aspartate aminotransferase (*n* = 11, 73 %). Treatment-related serious AEs were reported in 11 (73 %) patients. Nine patients (60 %) discontinued treatment due to drug-related toxicities. Immune-related AEs (irAEs) were reported in 14 patients (93 %). The most frequent irAEs were liver (*n* = 12, 80 %) and skin (*n* = 10, 67 %) toxicities. Five deaths were reported; all were caused by progressive disease. Efficacy evaluation showed one complete response, one partial response and four patients with stable disease. Best overall response rate was 13 % (2/15), and the disease control rate was 40 % (6/15). The study was terminated early due to frequent, high-grade liver toxicities.

**Conclusions:**

IPI 10 mg/kg plus DTIC 850 mg/m^2^ was not considered tolerable in the Japanese patient population.

ClinicalTrials.gov identifier: NCT01681212.

**Electronic supplementary material:**

The online version of this article (doi:10.1007/s00280-015-2870-0) contains supplementary material, which is available to authorized users.

## Introduction

Ipilimumab (IPI), a fully human IgG1 monoclonal antibody, is an antagonist to an immune-checkpoint receptor, cytotoxic T lymphocyte antigen-4 (CTLA-4), which downregulates antitumor T cell function [1, 2]. Blockade of CTLA-4 has been shown to increase effector T cell activation, proliferation and infiltration into tumors and to inhibit immunosuppressive T regulatory cell function and proliferation in tumor lesions [3–5].

IPI at a dose of 3 mg/kg has been approved by regulatory agencies in over 40 countries, including the USA [6] and recently in Japan, for the treatment of advanced (unresectable or metastatic) melanoma. A phase 3 study of previously treated patients with advanced melanoma showed significantly improved overall survival (OS) with IPI 3 mg/kg alone or IPI combined with gp100 compared to gp100 alone [7]. A small, open-label, phase 2 study of IPI 3 mg/kg monotherapy in Japanese patients with advanced melanoma showed similar safety and efficacy results as the trials of mostly Caucasian patients [8]. Results were similar in a phase 1 study of Japanese patients with advanced non-small cell lung cancer treated with IPI combined with paclitaxel/carboplatin [9]. Adverse events (AEs) with IPI were frequently immune-related, consistent with the drug’s mechanism of action. Management guidelines for immune-related AEs (irAEs) are available [10].

A pooled analysis of 10 prospective and 2 retrospective trials of IPI 3 or 10 mg/kg in advanced melanoma showed a 3-year survival rate of 22 % and a plateau in the survival curve beginning at approximately 3 years, with a follow-up to 10 years [11]. The pooled trials included patients receiving IPI monotherapy and those receiving combination therapies. One of the trials was a phase 3 study (CA184-024) of patients with untreated, advanced melanoma receiving either IPI 10 mg/kg plus dacarbazine (DTIC) 850 mg/m^2^ or DTIC plus placebo [12]. In that study, IPI plus DTIC resulted in a statistically significant improvement in OS compared with DTIC plus placebo. AEs were generally consistent with those seen in prior phase 2 studies of IPI monotherapy at a dose of 10 mg/kg [13–16]. However, the rates of Grade 3/4 hepatic events were higher [elevated alanine aminotransferase (ALT) level, 22 % in study CA184-024 versus 8 % in previous studies; elevated aspartate aminotransferase (AST) level, 18 % vs. 7 %], and the rates of Grade 3 or 4 gastrointestinal (GI) events were lower (rate of diarrhea, 4 % vs. 11 %; rate of colitis, 2 % vs. 5 %) than expected based on prior studies. The current study CA184-202 investigated the safety and efficacy of IPI 10 mg/kg plus DTIC 850 mg/m^2^ in Japanese patients with previously untreated advanced melanoma.

## Materials and methods

### Patients

The study recruited previously untreated Japanese patients with histologic diagnosis of stage III or IV melanoma according to TNM staging classification [17]. Prior adjuvant melanoma therapy was permitted. Key inclusion criteria were: life expectancy of ≥16 weeks; Eastern Cooperative Oncology Group (ECOG) performance status of 0 or 1; adequate liver, renal and bone marrow function by specified parameters; and age ≥20 years. Key exclusion criteria were: evidence of brain metastases; other malignancy within 5 years; history of or current active autoimmune disease; history of or concurrent GI perforations; human immunodeficiency virus, active hepatitis B, active hepatitis C or human T lymphotropic virus type 1 infection. Prohibited therapies included: prior use of any anticancer agent for melanoma, prior adjuvant therapy within 4 weeks of study drug administration, concomitant use of immunosuppressive agents or prior use of immunotherapy drugs, such as CTLA-4 inhibitors.

### Study oversight

This study was sponsored by Bristol-Myers Squibb. The study protocol was approved by the Institutional Review Board at each study site, and research was conducted in accordance with the standards specified by Article 14 Paragraph 3 and Article 80-2 of the Pharmaceutical Affairs Law, and Good Clinical Practice, as defined by the Ministerial Ordinance Concerning the Standards for the Implementation of Clinical Studies on Pharmaceutical Products and concerning notifications, and in accordance with ethical principles underlying the Declaration of Helsinki. All study participants provided written, informed consent prior to enrollment.

### Study design and treatment

**Fig. 1:**
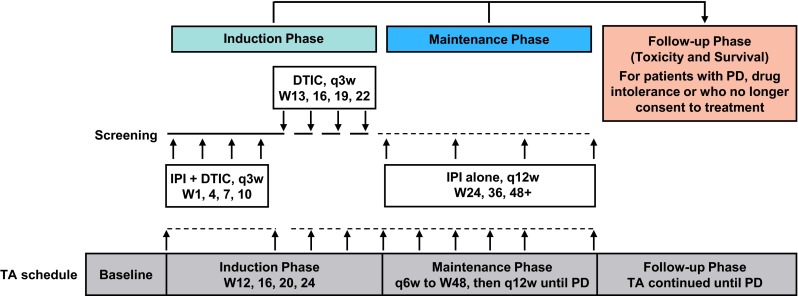
Schema showing 5 phases of study CA184-202 and the tumor assessment schedule. *DTIC* dacarbazine; *IPI* ipilimumab; *PD* progressive disease; *q3w* every 3 weeks; *q6w* every 6 weeks; *q12w* every 12 weeks; *TA* tumor assessment; *W* week

This was an open-label, single-arm, phase 2 study in previously untreated patients with unresectable or metastatic melanoma (ClinicalTrials.gov, NCT01681212) (Fig. 1). After screening for eligibility, patients entered the induction phase in which they received concomitant IPI 10 mg/kg and DTIC 850 mg/m^2^ every 3 weeks for four doses, both starting at week 1 (day 1), followed by 4 treatments of DTIC alone every 3 weeks. In the maintenance phase, patients without progressive disease who continued to tolerate treatment were administered IPI 10 mg/kg alone every 12 weeks. Tumor assessments were performed at weeks 12, 16, 20 and 24 during induction. During maintenance, tumor assessments were performed every 6 weeks until week 48 and then every 12 weeks until disease progression. Follow-up phases monitored toxicity, progressive disease and OS. Patients were followed for AEs for a minimum of 90 days following the last dose of IPI or for 30 days following the last dose of DTIC, whichever occurred later. AE assessments continued until all AEs were resolved, had returned to baseline or were deemed irreversible or stable.

### Study endpoints and assessments

The primary endpoint was survival rate at 1 year. The secondary endpoint was frequency of Grade 3/4 irAEs, reported in the following organ categories: GI, liver, skin, endocrine, neurological and other. irAEs were defined as drug-related and in a specified list from the most recent Medical Dictionary for Regulatory Activities (MedDRA) group terms and preferred terms. Toxicity management guidelines were provided by the sponsor (Supplemental Fig. 1) and have been published previously [10]. Exploratory endpoints included safety, OS, best overall response rate (BORR), progression-free survival (PFS), duration of response (DOR) and disease control rate (DCR).

Safety assessments were performed prior to study drug dosing, in addition to assessments made as part of the standard of care. Safety was evaluated using the National Cancer Institute Common Terminology Criteria for Adverse Events version 3.0. Response-based endpoints were captured using modified World Health Organization Criteria. Tumor assessment by computed tomography (CT) or magnetic resonance imaging (MRI) was required at screening; response was documented by CT or MRI methods similar to those used at screening.

### Statistical methods

Survival rate at 1 year was calculated from the number of patients alive at 1 year following the first dose of study therapy divided by the total number of patients treated, with a corresponding 2-sided 90 % confidence interval (CI). BORR and DCR were calculated with corresponding 2-sided 95 % CIs. Safety was evaluated in all treated patients. The study was designed so that with 26 patients there was an 80 % power to reject the null hypothesis that the true 1-year survival rate was ≤25 %.

## Results

### Patients, treatment and disposition

A total of 21 patients with advanced melanoma were enrolled in the study; 15 patients were treated with study drug [untreated patients either no longer met study inclusion criteria (*n* = 5) or were not treated due to administrative reasons (*n* = 1)]. Table 1 shows patient demographics. Most patients had M1b or M1c stage melanoma at screening, an ECOG performance status of 0 and normal lactate dehydrogenase levels. The majority of patients (60 %) had received prior adjuvant therapy. Most patients had normal baseline hematology results (≥73 %), liver function tests (≥93 %), renal function (creatinine, ≥93 %) and serum amylase and lipase (≥86 %).

**Table 1:** Patient demographics

Characteristic	Treated patients (*N* = 15)
Gender, *n* (%)	
Male	10 (66.7)
Race, *n* (%)	
Japanese	15 (100)
Age, years	
Median (range)	61 (36–70)
M stage at entry, *n* (%)	
M0	1 (6.7)
M1a	0 (0)
M1b	7 (46.7)
M1c	7 (46.7)
ECOG performance status	
0	13 (86.7)
1	2 (13.3)
Baseline LDH > ULN, *n* (%)	
Normal	12 (80)
Elevated	3 (20)
Prior adjuvant therapy	
Yes	9 (60)

*ECOG* Eastern Cooperative Oncology Group, *LDH* lactate dehydrogenase, *ULN* upper limit of normal

A total of 6 (40 %) patients received all four doses of IPI during the induction phase. Nine patients discontinued treatment prior to the fourth dose due to study drug toxicity or disease progression (*n* = 4 received three doses, *n* = 5 received two doses). The median number of doses per patient in the induction phase was 3 (range 2–4). Two patients (13 %) received all eight doses of DTIC during induction. Two patients were dosed in the maintenance phase, receiving one dose and three doses of IPI, respectively.

All 15 patients were off treatment at the conclusion of the study. Drug-related toxicity was the most common reason for study drug discontinuation [nine patients (60 %)]. Eight patients (53 %) discontinued due to elevations in serum ALT or AST levels. Five patients (33 %) discontinued due to disease progression, and one patient (7 %) had reached the maximum clinical benefit. The study was terminated early due to a high frequency of Grade 3/4 liver function test abnormalities following administration of IPI in combination with DTIC.

### Safety

Safety results are summarized in Table 2. A total of 14 (93 %) patients reported a serious adverse event (SAE) of any grade. There were 11 (73 %) patients with SAEs that were considered by the investigator to be drug-related (Grade 2–4 ALT and AST increases, Grade 2 colitis, Grade 3 diarrhea and Grade 1 dizziness) (Table 3). There were five deaths in this study, all of which were due to progressive disease, and all occurred more than 90 days after the last dose of study drug.

**Table 2:** Safety results summary

Event	Treated patients (*N* = 15)
AEs	
Grade 3/4 AEs	15 (100)
Drug-related AEs	15 (100)
Drug-related Grade 3/4 AEs	11 (73.3)
Most frequent AEs (occurring in ≥ 20 % of patients)	
ALT increased	12 (80)
AST increased	11 (73.3)
Constipation	8 (53.3)
Nausea	7 (46.7)
Rash	6 (40)
Diarrhea	5 (33.3)
Weight decreased	4 (26.7)
Back pain	4 (26.7)
Pyrexia	4 (26.7)
Decreased appetite	3 (20)
Diabetes mellitus	3 (20)
Hyperglycemia	3 (20)
Malignant neoplasm progression	3 (20)
irAEs	
Grade 3/4 irAEs	11 (73.3)
Skin irAEs	10 (66.7)
Liver irAEs	12 (80)
Gastrointestinal irAEs	6 (40)
Endocrine irAEs	3 (20)
Neurological irAEs	0 (0)
Other irAEs	2 (13.3)
SAEs	14 (93.3)
Drug-related SAEs	11 (73.3)
AEs leading to discontinuation of study therapy	9 (60)
Drug-related AEs leading to discontinuation	9 (60)

*AEs* adverse events, *ALT* alanine aminotransferase, *AST* aspartate aminotransferase, *irAEs* immune-related adverse events, *SAEs* serious adverse events

**Table 3:** On-study drug-related SAEs^a,b,c^

Organ category^a^	Treated patients (*N* = 15)				
	Worst grade, *n* (%)				
	Grade 1	Grade 2	Grade 3	Grade 4	Any grade
Any drug-related SAE	0	1 (6.7)	9 (60)	1 (6.7)	11 (73.3)
Investigations	0	0	9 (60)	1 (6.7)	10 (66.7)
ALT increased	0	0	9 (60)	1 (6.7)	10 (66.7)
AST increased	0	1 (6.7)	6 (40)	0	7 (46.7)
Gastrointestinal disorders	0	1 (6.7)	1 (6.7)	0	2 (13.3)
Colitis	0	1 (6.7)	0	0	1 (6.7)
Diarrhea	0	0	1 (6.7)	0	1 (6.7)
Nervous system disorders	1 (6.7)	0	0	0	1 (6.7)
Dizziness	1 (6.7)	0	0	0	1 (6.7)

*ALT* alanine aminotransferase, *AST* aspartate aminotransferase, *SAE* serious adverse event

^a^Patients may have had more than 1 event

^b^Drug-related events are those reported as related or missing

^c^On-study events are those reported between the first dose and 90 days after the last dose date of study treatment

All 15 subjects in this study reported at least 1 drug-related AE (Table 2). Nine (60 %) patients discontinued from the study due to drug-related Grade 2–3 AEs (Grade 3 ALT, AST increases; Grade 2 AST increase and Grade 2 colitis). The most common drug-related AE of any grade was ALT increase in 12 patients (80 %). There was 1 drug-related Grade 4 AE of elevation in ALT in 1 (6.7 %) patient, and there were no drug-related Grade 5 AEs.

The majority of drug-related AEs were immune-related. Grade 3/4 irAEs were reported in 11 (73 %) patients (Table 2). The most commonly reported irAEs were liver events (ALT increase and/or AST increase) in 12 patients (80 %). Eleven patients (73 %) experienced liver irAEs that were Grade 3 or 4 in severity. Skin irAEs were reported in 10 (67 %) patients, and GI irAEs were reported in 6 (40 %) patients. There were no GI perforations. Endocrine irAEs were reported in 3 (20 %) patients (*n* = 2 with Grade 2 hypothyroidism and *n* = 1 with Grade 2 hypophysitis). There were no neurological irAEs and no Grade 5 irAEs.

All patients who had irAEs (*n* = 14, 93 %) in this study were treated with symptomatic therapies, steroids and/or other immunosuppressive therapies for irAE management. All 14 patients were treated with corticosteroids. Nine patients were treated with immunosuppressive therapies. Eight patients received mycophenolic acid for liver toxicity, and one patient was treated with infliximab for diarrhea and colitis. All but 2 irAEs of ≥Grade 2 resolved to Grade 1 or less. Two patients with endocrine irAEs (hypophysitis or hypothyroidism) were unresolved, with continuation of hormone replacement therapy (L-thyroxine and levothyroxine) at study termination. A post-study irAE of autoimmune thyroiditis in the same patient with hypophysitis also remained ongoing, and treatment with L-thyroxine was continued. Time to onset of ≥Grade 2 irAEs ranged from 0.1 week (GI irAE) to 23 weeks (endocrine irAE) after initiation of study therapy. Among those that resolved, time to resolution ranged from 0.4 weeks (*n* = 3 GI irAEs) to 30 weeks (GI irAE).

### Efficacy

The primary endpoint of survival rate at 1 year could not be evaluated because the study was terminated early due to severe liver toxicity. However, survival rate at 1 year was observed to be 67 % (90 % CI 42.3, 85.8) at final database lock (Table 4). With one patient having a complete response that was ongoing at last contact and one patient achieving a partial response, the best overall response rate was 13 % (*n* = 2/15, 95 % CI 1.7, 40.5). Four additional patients had stable disease, making the disease control rate 40 % (*n* = 6/15, 95 % CI 16.3, 67.7) (Table 4).

**Table 4:** Overall survival and best overall response

Variable	Treated patients (*N* = 15)
Overall survival	
Survival rate at 1 year, *n* [% (90 % CI)]	10 [66.7 (42.3, 85.8)]
Best overall response	
Complete response, *n* (%)	1 (6.7)
Partial response, *n* (%)	1 (6.7)
Stable disease, *n* (%)	4 (26.7)
Progressive disease, *n* (%)	9 (60.0)
Best overall response rate, *n* [% (95 % CI)]	2 [13.3 (1.7, 40.5)]
Disease control rate, *n* [% (95 % CI)]	6 [40 (16.3, 67.7)]

*CI* confidence interval

## Discussion

This open-label, phase 2 study of IPI 10 mg/kg plus DTIC 850 mg/m^2^ in previously untreated Japanese patients with advanced melanoma was terminated early due to severe liver toxicity. Eleven out of 15 patients (73 %) had Grade 3/4 liver function test elevations considered to be immune-related, and 8 treatment discontinuations were due to elevations in ALT or AST. Other than hepatotoxicity, the safety profile in this trial was similar to previous experience with IPI. There were not enough patients to evaluate the primary efficacy endpoint. However, the observed survival rate at 1 year was 67 %.

In a phase 3 trial of approximately 500 previously untreated mostly Caucasian patients receiving IPI plus DTIC (in a similar treatment regimen to that of the present study) or DTIC plus placebo, the estimated survival rate with IPI plus DTIC was significantly greater than the rate with DTIC plus placebo at years 1 through 3 and at year 5 [12, 18]. However, the rates of immune-related Grade 3/4 elevations in ALT (21 %) and AST (17 %) reported in that study [12] were higher than Grade 3/4 hepatic irAEs previously reported in mostly Caucasian patients receiving IPI monotherapy at 3 or 10 mg/kg (0–12 %) [7, 15, 19]. The current study suggests that the risk of hepatic toxicity in Japanese patients is considerably higher. In a phase 1 trial of Japanese patients with non-small cell lung cancer, IPI 10 mg/kg in phased combination with paclitaxel and carboplatin was tolerable [20].

DTIC monotherapy is associated with hepatotoxicity [21–23], and the hepatotoxicity reported in the current study could have been due to the combination of IPI with DTIC. In a phase 2 study (CA184-396) of IPI 3 mg/kg monotherapy in Japanese patients, immune-related Grade 3 elevations in ALT and AST each occurred in only 5 % of patients, with no Grade 4 irAEs of any kind [8]. However, a high incidence of hepatotoxicity was also reported in a US phase 1 study of IPI combined with the *BRAF* mutation-targeted therapy vemurafenib [24]. This study was also terminated early due to liver toxicity. As in the present study, all hepatic AEs were reversible with either discontinuation of the study drugs or administration of corticosteroids or other immunosuppressant agents. Extensive global experience with IPI has enabled the development of effective treatment guidelines for the management of irAEs (Supplementary Fig. 1) [6].

Combination therapy of another immune-checkpoint inhibitor, nivolumab (NIVO), with IPI (IPI 3 mg/kg plus NIVO 1 mg/kg, q3w for four doses followed by NIVO 3 mg/kg q2w) demonstrated a significantly improved objective response rate compared with IPI therapy alone (61 % compared with 11 %, respectively) in *BRAF* wild-type patients treated in the phase 2 study CA209-069 [25]. Median PFS was not reached with NIVO plus IPI and was 4.4 months with IPI alone (hazard ratio 0.40; 95 % CI 0.23, 0.68, *p* < 0.001). Treatment-related hepatic AEs of Grade 3–4 occurred in 15 % of the predominantly Caucasian patients receiving the combination therapy. NIVO, approved in Japan for treatment of patients with advanced melanoma in the year 2014, is a fully human IgG4 monoclonal antibody against the programmed death-1 (PD-1) immune-checkpoint receptor, with a mechanism of action that is distinct from IPI [25].

In conclusion, in this study of Japanese patients with advanced melanoma, the incidence of severe liver toxicity was higher than experienced in other clinical studies. However, there were no new safety signals in Japanese patients in this study, and the overall safety profile was similar to prior experience, except for hepatotoxicity. IPI 10 mg/kg combined with DTIC 850 mg/m^2^ was not considered tolerable in this Japanese patient population. Due to early termination of the study, efficacy conclusions could not be reached. The observed rate of survival at 1 year was 67 %.

## Electronic supplementary material

Fig. 1 Management algorithms for IPI-related toxicities that affect the gastrointestinal tract (A), liver (B), skin (C) and endocrine system (D) (DOCX 465 kb)
