# Supplementary material for: Phase II study of the immune-checkpoint inhibitor ipilimumab plus dacarbazine in Japanese patients with previously untreated, unresectable or metastatic melanoma
Source: Cancer Chemother Pharmacol. 2015 Sep 25;76(5):969–75. doi: 10.1007/s00280-015-2870-0 (PMC4612320; doi:10.1007/s00280-015-2870-0)
Supplement: Supplementary file 1 — Management algorithms for IPI-related toxicities that affect the gastrointestinal tract (A), liver (B), skin (C) and endocrine system (D) (DOCX 465 kb) [file 280_2015_2870_MOESM1_ESM.docx]

**Supplementary Fig. 1** Toxicology management algorithms^a^

A.

B.

C.

D.

.

^a^Source: Bristol-Myers Squibb Company (2014) YERVOY® (ipilimumab) investigator brochure
